# Wnt1 Promotes EAAT2 Expression and Mediates the Protective Effects of Astrocytes on Dopaminergic Cells in Parkinson's Disease

**DOI:** 10.1155/2019/1247276

**Published:** 2019-09-09

**Authors:** Lei Wei, Chuan Chen, Li Ding, Mingshu Mo, Jing Zou, Zhenze Lu, Haiyan Li, Haotian Wu, Yongqiang Dai, Pingyi Xu, Zhengqi Lu

**Affiliations:** ^1^Department of Neurology, The Third Affiliated Hospital of Sun Yat-sen University, Guangzhou 510630, China; ^2^Department of Neurosurgery, The Third Affiliated Hospital of Sun Yat-sen University, Guangzhou 510630, China; ^3^Department of Pathology, The First Affiliated Hospital of Sun Yat-sen University, Guangzhou 510080, China; ^4^Department of Neurology, The First Affiliated Hospital of Guangzhou Medical University, Guangzhou 510120, China; ^5^Department of Neurology, The University of Hong Kong-Shenzhen Hospital, Shenzhen 518053, China

## Abstract

**Background:**

Wnt/*β*-catenin signaling has been reported to exert cytoprotective effects in a cellular model of Parkinson's disease (PD). Glutamate excitotoxicity has been suggested to contribute to the pathogenesis of PD, and excitatory amino acid transporters (EAATs) play a predominant role in clearing excessive glutamate. EAAT2 is mainly expressed in astrocytes, which are an important source of Wnt signaling in the brain.

**Methods:**

Wnt1-overexpressing U251 astrocytes were indirectly cocultured with dopaminergic SH-SY5Y cells treated with 6-hydroxydopamine (6-OHDA). Cell toxicity was determined by cell viability and flow cytometric detection. Glutamate level in the culture medium was determined by enzyme-linked immunosorbent assay (ELISA). Western blot analysis was used to detect the expression of Wnt1, *β*-catenin, and EAAT2. Immunofluorescence was used to display the expression and translocation of NF-*κ*B p65.

**Results:**

6-OHDA treatment significantly decreased cell viability in both U251 cells and SH-SY5Y cells, inhibited the expression of Wnt1, *β*-catenin, and EAAT2 in U251 cells, and increased the glutamate level in the culture medium. Coculture with Wnt1-overexpressing U251 cells attenuated 6-OHDA-induced apoptosis in SH-SY5Y cells. Overexpression of Wnt1 decreased the glutamate level in the culture media, upregulated *β*-catenin, EAAT2, and NF-*κ*B levels, and promoted the translocation of NF-*κ*B from the cytoplasm to the nucleus in U251 cells.

**Conclusion:**

Wnt1 promoted EAAT2 expression and mediated the cytoprotective effects of astrocytes on dopaminergic cells. NF-*κ*B might be involved in the regulation of EAAT2 by Wnt1.

## 1. Introduction

Parkinson's disease (PD), characterized by the loss of dopaminergic neurons in the substantia nigra (SN), is the second most common neurodegenerative disease after Alzheimer's disease in elderly people. The neuropathological features of PD are progressive loss of dopaminergic neurons in the SN and aggregation of *α*-synuclein-positive Lewy bodies in surviving neurons [1]. Although PD has been heavily researched over the last two decades, the precise etiology and pathogenesis of the disease remain not very clear. Studies have shown that several mechanisms are involved in the pathogenesis of PD, including oxidative stress, mitochondrial dysfunction, and elevated brain iron levels [2–4]. In addition, a wide range of studies have suggested that glutamate excitotoxicity contributes to the pathogenesis of PD and that excitatory amino acid transporters (EAATs) play a predominant role in clearing the excessive glutamate in the synaptic cleft [5]. EAAT2 and EAAT1 (glutamate transporter 1 (GLT-1) and glutamate-aspartate transporter (GLAST) in rodents, respectively) are the main transporters that maintain optimal glutamate levels in the synaptic clefts [6]. Reduced expression and function of these transporters, especially EAAT2, have been reported in numerous neurodegenerative disorders, including amyotrophic lateral sclerosis, Alzheimer's disease, and PD [6–8].

Wnt signaling pathway is an autocrine-paracrine signal transduction pathway that has been demonstrated to participate in embryonic development, cell differentiation, and ontogenesis [9–11]. A main Wnt signaling pathway branch is the Wnt/*β*-catenin pathway, in which Wnt proteins bind to Frizzled receptors and to initiate the activation of Dishevelled [12]. The activation of Dishevelled results in the inhibition of glycogen synthase kinase-3*β* (GSK3*β*), which in turn stabilizes *β*-catenin [12]. Stabilized *β*-catenin accumulates and is taken into the nucleus where it regulates the expression of numerous genes [12]. Extensive research has confirmed the vital role of Wnt signaling in midbrain dopaminergic neuronal development. For example, Wnt1 and Wnt3a, which exert effects by the Wnt/*β*-catenin pathway, are key regulators in the development of dopaminergic neurons [13]. Cellular protective effects of the Wnt/*β*-catenin pathway have been demonstrated in animal and cellular models of Alzheimer's disease, retinal degeneration, cerebral ischemia, and PD [14–17]. Our previous studies demonstrated that the activation of the Wnt/*β*-catenin signaling pathway by exogenous Wnt1 or Wnt3a protects cells by restoring mitochondria function [18, 19].

Astrocytes are the most abundant glial cell type in the brain and exert protective effects on neurons. Impairment in astrocyte function can critically influence neuronal survival and lead to neurodegeneration [20]. Several studies have shown that astrocytes were able to express specific Wnt ligands, receptors, and regulators under physiological and pathological conditions, indicating the involvement of this family of proteins in astroglial functioning in the normal and damaged central nervous system [21–24]. Moreover, studies have shown that Wnt signaling could induce the expression of EAAT2 which is mainly expressed in astrocytes [25, 26]. Here, we cocultured dopaminergic SH-SY5Y cells and U251 astrocytes overexpressing Wnt1 and found that Wnt1 promoted the expression of EAAT2 in astrocytes and mediated the protective effects of astrocytes on dopaminergic cells.

## 2. Materials and Methods

### 2.1. Cell Culture

Human neuroblastoma SH-SY5Y cells and U251 astrocytes were obtained from the American Type Culture Collection (ATCC, Manassas, VA, U.S.A.), maintained in DMEM with high glucose (Invitrogen, USA) supplemented with 10% fetal bovine serum (FBS, Invitrogen), and cultured in a humidified incubator with 5% CO_2_ at 37°C. Cells with 20-30 passages were used. For experiments, cells were seeded at a density of 1 × 10^5^/cm^2^ in plastic flasks or plates. Cells were treated with different concentrations of 6-hydroxydopamine (6-OHDA, Sigma-Aldrich, USA) according to the corresponding experiments and with vehicle as a control. To block the receptor of the Wnt signal, a Frizzled-1 antagonist, human recombinant Dickkopf- (DKK-) 1 protein (Sigma-Aldrich, USA), or vehicle was added to the cultures 10 min prior to 6-OHDA.

### 2.2. Transfection of Plasmid pCDNA3.1-Wnt1

The Wnt1 expression vector (pCDNA3.1-Wnt1) was constructed by Forevergen company (Guangzhou, China) using human nephridial tissue complimentary DNA (cDNA) as the template and confirmed by sequencing. pCDNA3.1-Wnt1 was transfected into U251 cells (U251-Wnt1), while the pCDNA3.1 empty vector was transfected as a control (U251-EV). All cell transfections were conducted using Lipofectamine 2000 reagent (Invitrogen, USA) following the manufacturer's protocol. Quantitative PCR and Western blot assays were used to identify the expression level of Wnt1.

### 2.3. Real-Time PCR

Forty-eight hours after transfection, total RNA was isolated using TRIzol (Invitrogen) according to the manufacturer's protocol. Then, 1 *μ*g of total RNA was reverse transcribed into first-strand cDNA using a reverse transcription (RT) kit (Takara, Japan) according to the manufacturer's protocol in a final volume of 20 *μ*l. For analysis of Wnt1 expression with quantitative real-time RT-polymerase chain reaction (RT-PCR), human glyceraldehyde-3-phosphate dehydrogenase (GAPDH) was used as an internal control, and the sequences of the primers were as follows: Wnt1 forward: 5′-GGTTCCATCGAATCCTGCAC-3′ and Wnt1 reverse: 5′-GCCTCGTTGTTGTGAAGGTT-3′; GAPDH forward: 5′-GAAGGTGAAGGTCGGAGTC-3′ and GAPDH reverse: 5′-GAAGATGGTGATGGGATTTC-3′. One microliter of the synthesized cDNA was used in real-time PCR together with SYBR Green I Master Mix (SYBR® Premix Ex Taq™, Takara, Japan) on an MJ Research Opticon2 real-time thermocycler (Bio-Rad, Hercules, CA, USA). Fluorescent reading from the real-time PCR was quantitatively analyzed by determining the difference in Ct (delta Ct) between Ct of Wnt1 and its internal GAPDH, and Wnt1 gene expression was determined by the formula 2^−deltaCt^.

### 2.4. Indirect Astrocyte-Neuron Coculture System

SH-SY5Y cells and U251 cells were cocultured in 6-well Transwell Permeable Supports (polystyrene, pore size, 0.4 *μ*m, Corning Company, USA) where cells shared medium coming into contact with one another. U251 cells were seeded on Transwell inserts, and SH-SY5Y cells were cultured in the lower compartment. Cells were divided into 6 groups: SH-SY5Y cells (control), SH-SY5Y cells treated with 50 *μ*M 6-OHDA, SH-SY5Y cells cocultured with U251-Wnt1 cells and treated with 50 *μ*M 6-OHDA, SH-SY5Y cells cocultured with U251-Wnt1 cells and treated with 50 *μ*M 6-OHDA and 100 ng/ml DKK-1, and SH-SY5Y cells cocultured with U251-EV and treated with 50 *μ*M 6-OHDA.

### 2.5. Cell Viability Assay

Cell viability was determined by colorimetric MTT (3-(4,5-dimethylthiazol-2-yl)-2,5-diphenyltetrazoliumbromide, Sigma) assay [27]. After treatment, SH-SY5Y cells or U251 cells were seeded in a 96-well plate at a density of 1 × 10^3^ cells per well. After attachment, cells were incubated with 0.5 mg/ml MTT for 4 h at 37°C. Following aspiration of the MTT solution, the same volume of DMSO was added into each well to dissolve the purple formazan crystals. Absorbance was read in a microtiter plate reader at 490 nm. Cell viability was expressed as a percentage of the absorbance of control cells.

### 2.6. Flow Cytometric Detection of Apoptotic Cells

SH-SY5Y cell apoptosis was quantified by flow cytometry using fluorescein isothiocyanate- (FITC-) conjugated annexin V and propidium iodide (PI). The cells were seeded in 6-well Transwell Permeable Supports (5 × 10^5^ cells/well). After the treatment described above, the cells were harvested, washed with cold PBS, and double-stained using an annexin V-FITC apoptosis detection kit (KeyGen, China). According to the manufacturer's instructions, cells resuspended in annexin V-FITC binding buffer were incubated with annexin V-FITC and PI for 10 min at room temperature in the dark. The number of cells under apoptosis and necrosis was evaluated by flow cytometry (BD, CA, USA). At least 10,000 cells were analyzed.

### 2.7. Glutamate Measurement

The concentrations of glutamate in the culture media were measured using an enzyme-linked immunosorbent assay (ELISA) kit (Qincheng Biotechnology Company, China). The cells were seeded in 6-well Transwell Permeable Supports (5 × 10^5^ cells/well). After the treatment described above, the conditioned media were collected and then centrifuged at 1,000 × RPM for 10 min to get rid of impurities. According to the manufacturer's instructions, 10 *μ*l culture medium of each group was used for measurement. Absorbance was read in a microplate reader at 450 nm. The glutamate levels were expressed as the percentage versus the control group.

### 2.8. Western Blot Assay

Immunoblotting was performed in accordance with a standard procedure as described before [27]. Briefly, U251 cells in 6-well Transwell Permeable Supports were washed twice with prechilled PBS followed by lysis with the Mammalian Cell Extraction Kit (Biovision, CA, USA) mixed with PhosSTOP (Roche, Basel, Switzerland). After the protein concentrations were measured with the BCA protein assay kit (Thermo Fisher Scientific Inc., IL, USA), cell lysates were separated by 8%~12% SDS-polyacrylamide gels and then transferred onto polyvinylidene difluoride (PVDF) membranes (Millipore, MA, USA). After transfer, the membranes were blocked with 5% nonfat dry milk (Sigma, USA) in Tris-buffered saline (20 mM Tris-HCl pH 7.6, 137 mM NaCl) containing 0.01% Tween 20 (TBST) for 1 h at room temperature, and then, the membranes were probed with primary antibodies diluted according to the producers' datasheet at 4°C overnight. The following primary antibodies were used: rabbit anti-*β*-catenin (1 : 1,000 dilution, Abcam, Cambridge, UK), rabbit anti-EAAT2 (1 : 1,000 dilution, CST, USA), mouse anti-*β*-actin (1 : 1,000 dilution, Millipore, USA), and rabbit anti-Wnt1 (1 : 800 dilution, Abcam, Cambridge, UK). Subsequently, membranes were washed with TBST 3 times followed by incubation with HRP-labeled anti-mouse IgG or anti-rabbit IgG (KPL, MD, USA) secondary antibodies at room temperature for 1 h. After 3 washes with TBST, proteins were detected with the SuperSignal® West Pico Chemiluminescent Substrate (Thermo Fisher Scientific Inc., IL, USA), and membranes were exposed to X-ray films (Fujifilm Corporation, Japan), which were scanned and analyzed using Quantity One v4.62 for Windows software (Bio-Rad, CA, USA).

### 2.9. Immunofluorescence Staining

U251 cells, U251-Wnt1 cells, or U251-EV cells were treated with vehicle, 6-OHDA (50 *μ*M), or/and DKK-1 (100 ng/ml) for 24 h, followed by immunofluorescent staining as described before [27]. Briefly, cells were washed three times with PBS and fixed in 4% paraformaldehyde-PBS for 15 min at room temperature. The fixed cells were permeabilized with 0.01 M PBS containing 0.1% Triton X-100 for 20 min and then blocked with 10% goat serum for 1 h at room temperature. After blocking, cells were incubated overnight at 4°C with primary antibodies diluted in 10% goat serum/rabbit anti-NF-*κ*B p65 (1 : 800, CST, USA). Cells were washed three times with 0.01 M PBS for 5 min. Alexa Fluor 555-conjugated goat anti-rabbit antibody (CST, USA) was used as a secondary antibody at a dilution of 1 : 1,000 and incubated for 2 h at room temperature. Nuclei were stained with 4′,6-diamidino-2-phenylindole (DAPI, CST, USA) for 45 s at room temperature before cells were examined under a confocal laser scanning microscope LEICA TCS SP5 MP (Leica, Heidelberg, Germany).

### 2.10. Statistical Analysis

The results are presented as the mean ± standard deviation (SD). Differences among various time points or groups were examined by using analysis of variance (ANOVA) followed by Tukey-Kramer tests for post hoc multiple comparisons. We also used Student's *t*-test for pairwise comparisons at a given treatment. The level of significance was set at *P* < 0.05. All statistical analyses were performed with SPSS 12.0 for Windows (SPSS Inc., Chicago, IL, USA).

## 3. Results

### 3.1. Cytotoxic Effect of 6-OHDA on U251 Cells

**Figure 1:**
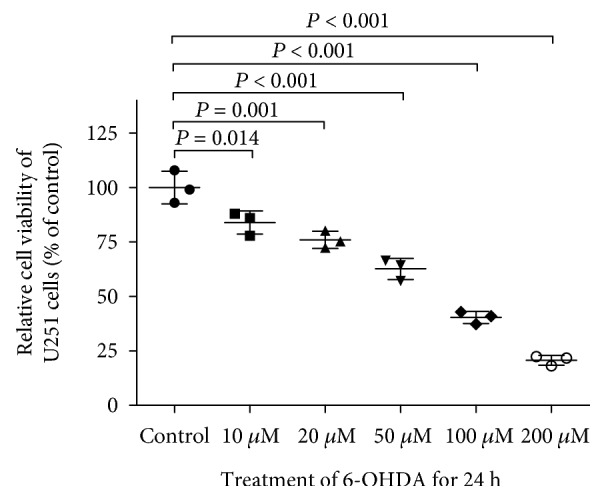
Cytotoxic effect of 6-OHDA on U251 cells. U251 cells were treated with 0-200 *μ*M 6-OHDA for 24 h, and cell viability was measured by MTT assay and expressed as a percentage of that in the vehicle group. All of the data are presented as the mean ± SD from 3 independent experiments. Differences among groups were examined by using ANOVA followed by Tukey-Kramer tests for post hoc multiple comparisons. *F* value = 112.6, degrees of freedom between groups (df_between groups_) = 5, degree of freedom within groups (df_within groups_) = 12, and the total degree of freedom (df_total_) = 17.

Cell viability was measured after 0-200 *μ*M 6-OHDA treatment for 24 h and showed a concentration-dependent reduction in cell viability. Compared with the cell viability of control cells, cell viability was 83.97 ± 3.07% with 10 *μ*M 6-OHDA, 75.97 ± 2.26% with 20 *μ*M, 62.65 ± 2.82% with 50 *μ*M, 40.37 ± 1.62% with 100 *μ*M, and 20.70 ± 1.31% with 200 *μ*M (Figure 1), and 50 *μ*M 6-OHDA was chosen for further experiments.

### 3.2. 6-OHDA Treatment Caused Downregulation of Wnt1 in U251 Cells

**Figure 2:**
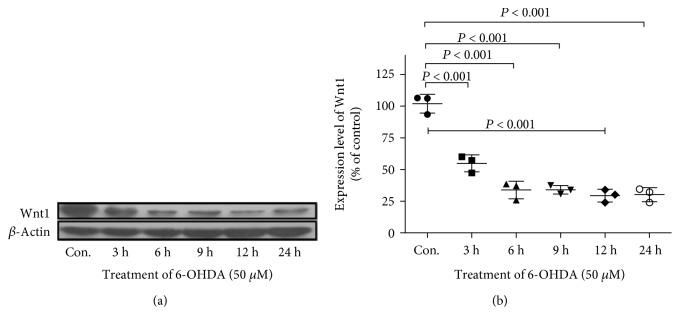
6-OHDA treatment downregulated the protein level of Wnt1 in U251 cells. (a) U251 cells were treated with vehicle or 6-OHDA (50 *μ*M) for 3, 6, 9, 12, and 24 h, and the protein level of Wnt1 was detected by Western blot with *β*-actin as an internal control. (b) The relative band intensities of Wnt1 were measured by Quantity One software and normalized to the expression of *β*-actin in U251 cells. All of the data are presented as the mean ± SD from three independent experiments. Differences among various time points were examined by using ANOVA followed by Tukey-Kramer tests for post hoc multiple comparisons. *F* value = 67.10, df_between groups_ = 5, df_within groups_ = 12, and df_total_ = 17.

6-OHDA is an endogenous oxidative metabolite of dopamine commonly used to generate experimental models of PD. A previous study demonstrated that the Wnt signal in the central nervous system mainly originates from astrocytes [21]. U251 astrocyte cells were used to study the change in Wnt1 expression after 6-OHDA treatment. U251 cells were treated with 50 *μ*M 6-OHDA for 3, 6, 9, 12, and 24 h. Then, total protein was extracted, and the level of Wnt1 was determined by Western blot (Figure 2). 6-OHDA treatment caused a time-dependent decrease in Wnt1. Compared to Wnt1 expression in the control, the Wnt1 level decreased to 54.82 ± 6.69% at 3 h, 33.84 ± 4.00% at 6 h, and remained relatively stable from 9 h to 24 h.

### 3.3. Overexpression of Wnt1 in U251 Cells

**Figure 3:**
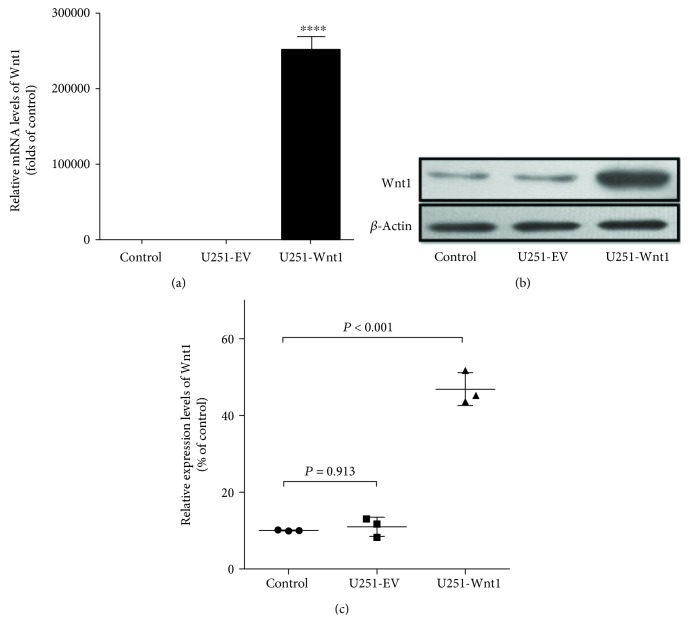
Evaluation of the Wnt1 overexpression vector in U251 cells by Western blot and PCR. Forty-eight hours after transfection with the Wnt1 expression vector or empty vector, U251 cells were harvested, and the mRNA and protein levels of Wnt1 were determined by real-time PCR and Western blot. The mRNA (a) and protein (b, c) levels of Wnt1 were significantly higher in U251 cells overexpressing Wnt1 than in control cells, whereas the empty vector did not influence Wnt1 expression. ^∗∗∗∗^*P* < 0.0001. For Western blot assay, differences among groups were examined by using ANOVA followed by Tukey-Kramer tests for post hoc multiple comparisons (c). *F* value = 161.42, df_between groups_ = 2, df_within groups_ = 6, and df_total_ = 8.

To investigate the effects of Wnt1 in astrocytes, we constructed U251 cell lines overexpressing Wnt1. As shown in Figure 1, the mRNA (Figure 3(a)) and protein (Figure 3(b)) levels of Wnt1 were significantly higher in U251 cells transfected with the Wnt1 expression vector than in control cells, while cells transfected with the empty vector did not show a change in Wnt1 mRNA and protein expression.

### 3.4. Coculturing with U251-Wnt1 Cells Attenuated 6-OHDA-Induced SH-SY5Y Cell Injury

**Figure 4:**
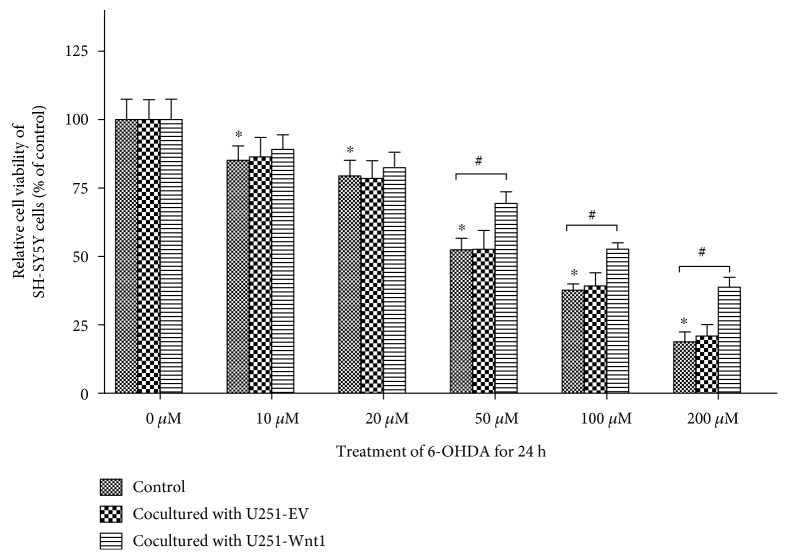
Coculturing with U251-Wnt1 cells attenuated 6-OHDA-induced injury in SH-SY5Y cells. SH-SY5Y cells were indirectly cocultured with U251-Wnt1 cells or U251-EV cells, with SH-SY5Y cells cultured alone as the control, and then treated with 50 *μ*M 6-OHDA for 24 h; cell viability was assessed using the MTT assay. The data are expressed as percentages relative to the control group with 0 *μ*M 6-OHDA and are presented as the mean ± SD from four independent experiments. ^∗^*P* < 0.05 compared to the control with 0 *μ*M 6-OHDA; ^#^*P* < 0.05 compared to the control group at the corresponding 6-OHDA concentration.

Treatment with 6-OHDA for 24 h caused a concentration-dependent reduction in cell viability in SH-SY5Y cells (Figure 4). Compared with the cell viability of controls, cell viability was 85.12 ± 5.31% with 10 *μ*M 6-OHDA, 79.43 ± 5.69% with 20 *μ*M, 52.41 ± 4.23% with 50 *μ*M, 37.67 ± 2.34% with 100 *μ*M, and 18.77 ± 3.61% with 200 *μ*M.

When cocultured with U251-EV cells or U251-Wnt1 cells, treatment with 6-OHDA for 24 h also caused a concentration-dependent reduction in cell viability in SH-SY5Y cells (Figure 4). Coculturing with U251-EV cells did not change SH-SY5Y cell viability. When cocultured with U251-Wnt1, the cell viability of SH-SY5Y cells was significantly higher than that of the isolated SH-SY5Y cells after treatment with 50 *μ*M, 100 *μ*M, and 200 *μ*M 6-OHDA.

**Figure 5:**
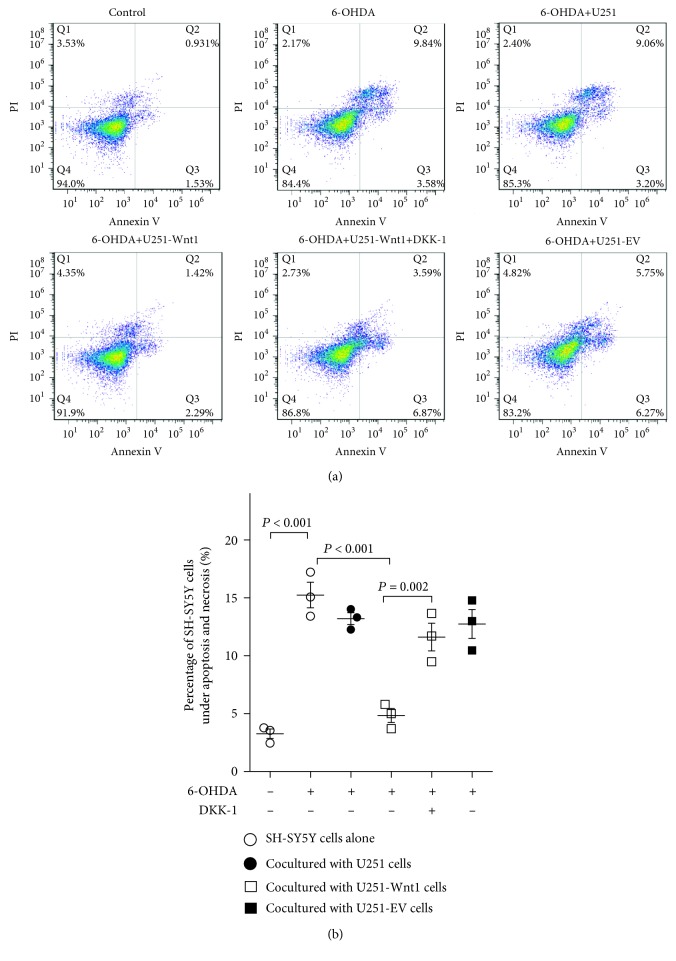
Effect of coculturing with U251-Wnt1 cells on 6-OHDA-induced damage in SH-SY5Y cells measured by flow cytometry. SH-SY5Y cells were indirectly cocultured with U251 cells, U251-Wnt1 cells, or U251-EV cells and then treated with or without 50 *μ*M 6-OHDA or 100 ng/ml DKK-1 for 24 h, and a combination of annexin V-FITC and PI staining was performed followed by flow cytometry. (a) Representative set of flow cytometric two-parameter dot plots in the corresponding groups. The lower right quadrant, which shows annexin V-FITC-positive and PI-negative cells, represents early apoptotic cells. The upper right quadrant, which shows both annexin V-FITC- and PI-positive cells, represents cells in the end stages of apoptosis and necrosis. (b) The percentage of cells under apoptosis and necrosis. Data are presented as the mean ± SD from 3 independent experiments. Differences among groups were examined by using ANOVA followed by Tukey-Kramer tests for post hoc multiple comparisons. *F* value = 28.59, df_between groups_ = 5, df_within groups_ = 12, and df_total_ = 17.

Then, we further examined the presence of cells under apoptosis and necrosis using the FITC-annexin-V/PI staining assay (Figure 5). Annexin V has a strong affinity for phosphatidylserine that translocates from the inner surface of the plasma membrane to the cell surface upon initiation of apoptosis and is widely used as a marker of apoptosis. When used in combination with PI, apoptotic cells can easily be differentiated from necrotic and living cells. The percentage of cells under apoptosis and necrosis in the control group was 3.27 ± 0.71%, while that in 6-OHDA-treated cells was 15.24 ± 1.90%. However, coculturing with U251-Wnt1 cells reduced the 6-OHDA-induced injury to 4.84 ± 0.61%, and this reduction was partly blocked by 100 ng/ml DKK-1, an antagonist of the Wnt/*β*-catenin pathway. Coculturing with U251 cells or U251-EV cells did not significantly reduce 6-OHDA-induced SH-SY5Y cell injury.

### 3.5. Wnt1 Overexpression Decreased the Glutamate Level in Culture Medium

**Figure 6:**
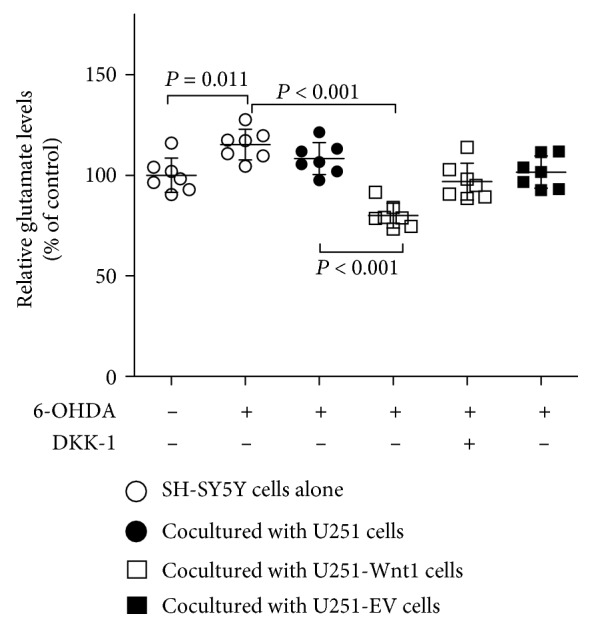
Wnt1 overexpression decreased the glutamate level in culture medium. SH-SY5Y cells were indirectly cocultured with U251 cells, U251-Wnt1 cells, or U251-EV cells and then treated with or without 50 *μ*M 6-OHDA or 100 ng/ml DKK-1 for 24 h, and the glutamate levels in culture media were detected by ELISA. The data are expressed as percentages relative to the control group. All of the data are presented as the mean ± SD from 7 independent experiments. Differences among groups were examined by using ANOVA followed by Tukey-Kramer tests for post hoc multiple comparisons. *F* value = 15.87, df_between groups_ = 5, df_within groups_ = 36, and df_total_ = 41.

To confirm the effect of Wnt1 overexpression on the toxicity of excitatory amino acids, the glutamate level in culture medium was detected. Treatment with 50 *μ*M 6-OHDA for 24 h significantly increased the glutamate level in the culture medium of SH-SY5Y cells to 115.28 ± 7.62% of that in the control group (*P* = 0.011). Coculturing with U251-Wnt1 cells could decrease the glutamate level to 79.97 ± 6.16%, which could be blocked by the antagonist of Wnt signaling, DKK-1 (Figure 6).

### 3.6. Wnt1 Overexpression Upregulated EAAT2 Expression

**Figure 7:**
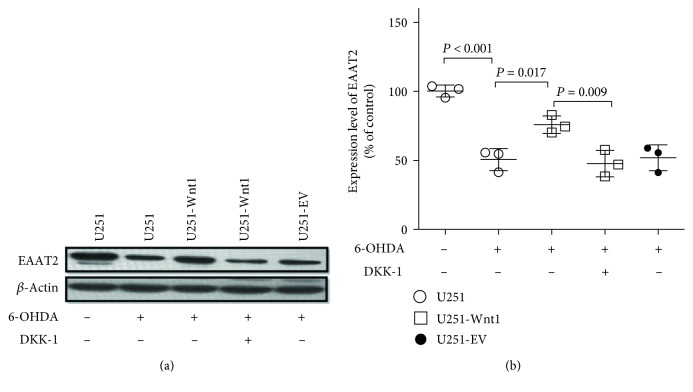
Wnt1 overexpression attenuated the inhibitory effect of 6-OHDA on EAAT2 expression in U251 cells. U251 cells, U251-Wnt1 cells, or U251-EV cells were treated with vehicle, 6-OHDA (50 *μ*M), or/and DKK-1 (100 ng/ml) for 24 h, and the protein levels of EAAT2 (a) were detected by Western blot with *β*-actin as an internal control. The relative band intensities of EAAT2 (b) were measured by Quantity One software and normalized to the expression of *β*-actin. Differences among groups were examined using ANOVA followed by using Tukey-Kramer tests for post hoc multiple comparisons. Data are presented as the mean ± SD of 3 experiments. *F* value = 25.29, df_between groups_ = 4, df_within groups_ = 10, and df_total_ = 14.

Reduced expression of EAAT2 has been reported in PD [28]. Here, Western blotting was used to test the effects of 6-OHDA and/or Wnt1 overexpression on EAAT2 levels in U251 cells (Figure 7). Treatment with 50 *μ*M 6-OHDA for 24 h decreased the protein level of EAAT2 in U251 cells to 50.57 ± 7.98% of the protein level of EAAT2 in the control. However, the protein level of EAAT2 in U251-Wnt1 cells was increased to 75.84 ± 6.39% of the control level after 6-OHDA treatment, which indicated that the overexpression of Wnt1 attenuated the inhibitory effect of 6-OHDA on EAAT2 expression. The effect of Wnt1 upregulation on EAAT2 expression could be partly blocked by DKK-1.

### 3.7. Wnt1 Overexpression Activated the Wnt/*β*-Catenin Pathway

**Figure 8:**
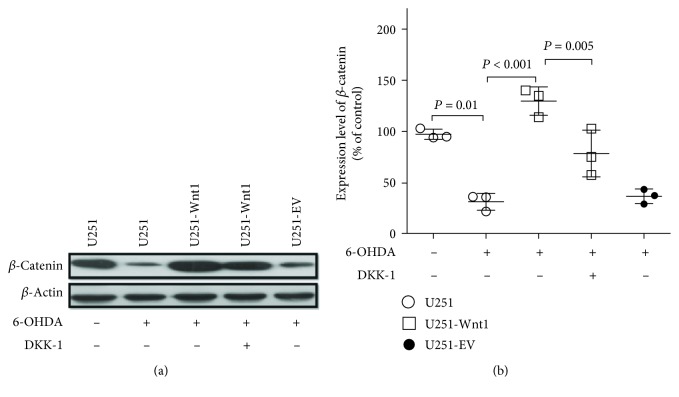
Wnt1 overexpression activated the Wnt/*β*-catenin pathway in U251 cells. U251 cells, U251-Wnt1 cells, or U251-EV cells were treated with vehicle, 6-OHDA (50 *μ*M), or/and DKK-1 (100 ng/ml) for 24 h, and the protein levels of *β*-catenin (a) were detected by Western blot with *β*-actin as an internal control. The relative band intensities of *β*-catenin (b) were measured by Quantity One software and normalized to the expression of *β*-actin. Differences among groups were examined using ANOVA followed by using Tukey-Kramer tests for post hoc multiple comparisons. Data are presented as the mean ± SD of 3 experiments. *F* value = 30.23, df_between groups_ = 4, df_within groups_ = 10, and df_total_ = 14.

To confirm the activation effect of endogenous Wnt1 overexpression on the Wnt/*β*-catenin pathway, Western blotting was used to detect the expression of *β*-catenin in U251 cells. Treatment with 50 *μ*M 6-OHDA for 24 h decreased the *β*-catenin level to 32.36 ± 5.93% of that in control cells, while overexpression of Wnt1 reversed the inhibitory effect of 6-OHDA treatment on *β*-catenin expression to 129.6 ± 7.99% of the control level. This reversal effect of Wnt1 overexpression on *β*-catenin expression was blocked by DKK-1, indicating that endogenous Wnt1 might exert effects via the autocrine pathway (Figure 8).

### 3.8. Wnt1 Overexpression Activated the NF-*κ*B Signaling Pathway

**Figure 9:**
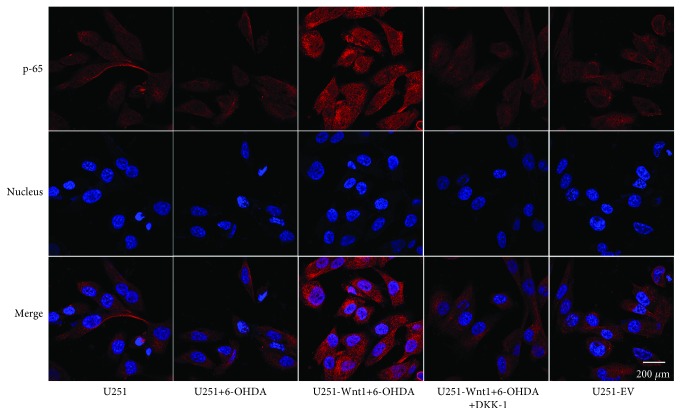
Immunofluorescence staining of p65 protein in U251 cells. U251 cells, U251-Wnt1 cells, or U251-EV cells were treated with vehicle, 6-OHDA (50 *μ*M), or/and DKK-1 (100 ng/ml) for 24 h, and the expression and distribution of p65 were examined using an antibody against p65 (red). Nuclei were counterstained with DAPI (blue). All images were captured using a confocal laser scanning microscope.

Previous studies have demonstrated that NF-*κ*B contributes to the neuron-dependent induction of GLT-1 (the rodent homologue for EAAT2) expression [29]. To determine whether NF-*κ*B participates in the regulation of Wnt1 on EAAT2 expression, p65 and DAPI double staining was assessed. We found a small amount of expression of NF-*κ*B p65 in the cytoplasm and nucleus of U251 cells with or without 6-OHDA treatment, whereas Wnt1 overexpression caused an upregulation in expression both in the cytoplasm and the nucleus, indicative of NF-*κ*B signaling pathway activation (Figure 9).

## 4. Discussion

The study investigated the protective effects of astrocytes on dopaminergic cells mediated by Wnt signaling. Our present study demonstrated that Wnt1 levels in U251 cells were decreased after 6-OHDA treatment and that coculture with Wnt1-overexpressing U251 astrocytes attenuated the toxic effect of 6-OHDA on dopaminergic SH-SY5Y cells. Coculture with Wnt1-overexpressing U251 astrocytes also could decrease the glutamate level in the culture medium. Moreover, the Wnt1-mediated protective effects of astrocytes on dopaminergic cells might activate the NF-*κ*B signaling pathway and upregulate the expression of EAAT2.

We cocultured SH-SY5Y cells with U251 astrocytes to simulate an *in vivo* environment. Both SH-SY5Y and U251 are human cell lines. The SH-SY5Y cell line was chosen in this study for its expression of tyrosine hydroxylase, which leads to its consideration as a dopaminergic cell line used to simulate dopaminergic neurons [30]. As an endogenous oxidative metabolite of dopamine, 6-OHDA has been found to be taken up by the plasma membrane dopamine transporter. Once in the cytoplasm, the cytotoxicity of 6-OHDA has been thought to be based primarily on dopaminergic neuron damage by mechanisms similar to those that have been proposed for patients with PD. For example, 6-OHDA inhibits mitochondrial complex I, produces large amounts of free radicals, induces cell death, and has been widely used to study the neurodegenerative process in PD [31, 32]. It has also been shown that 6-OHDA induces apoptosis in various cell types that do not express dopaminergic transporters, such as PC12 cells and astrocytes [33–35]. For example, Gupta et al. reported a significantly decreased mitochondrial dehydrogenase activity, mitochondrial membrane potential, augmented reactive oxygen species (ROS) level, caspase-3 mRNA level, and chromatin condensation, and DNA damage was observed in 6-OHDA-treated astrocytes [33]. Kaddour et al. also reported that 6-OHDA treatment caused cell death in cultured rat astrocytes through enhancing the level of ROS, associated with a reduction of both superoxide dismutase and catalase activity, and exposure of cultured astrocytes to 6-OHDA concentrations above 30 *μ*M reduced astrocyte viability in a concentration- and time-dependent manner [34]. A higher concentration of 6-OHDA may increase the permeability of the cell membrane, enter the cytoplasm, and play a toxic role.

Wnt1 is a cysteine-rich glycosylated protein that promotes neuronal cell and astrocyte crosstalk as a mechanism of neuroprotection in PD models [21]. The involved neuroprotective effect may consist of two aspects. As demonstrated by previous studies, Wnt1 is secreted by astrocytes, binds to Frizzled receptors, and exerts a direct protective effect on dopaminergic neurons. For example, a recent study by L'Episcopo et al. showed that exogenous Wnt1 exerts robust neuroprotective effects against caspase-3 activation, loss of tyrosine hydroxylase-positive neurons, and ^[3H]^dopamine uptake induced by DA-specific insults, including serum and growth factor deprivation, 6-OHDA and 1-methyl-4-phenyl-1,2,4,5-tetrahydropyridine (MPTP)/the 1-methyl-4-phenylpyridinium ion (MPP+) [22]. Our previous study also found that exogenous Wnt1 could increase the *β*-catenin protein level, potentially contributing to the protection of SH-SY5Y cells against 6-OHDA toxicity by inhibiting mitochondrial stress and endoplasmic reticulum stress [18]. On the other hand, Wnt1 might also bind to Frizzled receptors on astrocytes by autocrine effects and regulate the protective effects of astrocytes on dopaminergic neurons (indirect protective effect). In the present study, we found that Wnt1 regulated the EAAT2 level in astrocytes and protected dopaminergic cells against 6-OHDA toxicity.

Glutamate excitotoxicity has been shown to participate in the pathogenesis of PD. For example, Li and colleagues found higher levels of glutamate both in the cerebrospinal fluid and the striatum in a 6-OHDA-induced PD model [36]. Excessive glutamate in the synaptic cleft overstimulates the ionotropic and metabotropic glutamate receptors in the postsynaptic membrane and mediates excitotoxicity [37]. Previous studies have revealed that glutamate excitotoxicity induces dopamine neuron death, movement disorder, and cognitive impairment; thus, glutamate excitotoxicity plays an important role in the pathogenesis of PD [37–39]. Glutamate transporters prevent the overstimulation of postsynaptic glutamate receptors that leads to excitotoxic neuronal injury. To date, five subtypes of glutamate transporters have been identified. EAAT1 and EAAT2 are predominantly expressed in astrocytes and are responsible for the uptake of excess glutamate from the extracellular space [6, 37]. In the adult brain, EAAT2 accounts for >90% of extracellular glutamate clearance [6]. Impaired glutamate uptake and reduced EAAT2 expression are found in PD animal models constructed by 6-OHDA or MPTP [28, 38, 39]. Our present study also showed that treatment with 6-OHDA caused a decrease in EAAT2 expression and an increase of glutamate level in the culture medium.

A previous study showed that Wnt1 could induce expression of GLT-1 (the rodent homologue for EAAT2) in rat C6 glioma cells [26]. Here, we also obtained a similar result, showing that Wnt1 overexpression increased EAAT2 levels in U251 astrocytes. Then, increased EAAT2 level attenuated the increase of glutamate level in the culture medium and consequently reduced the toxicity of excitatory amino acids. Previous studies have shown that the NF-*κ*B pathway is critical for positive transcription of EAAT2. NF-*κ*B binds to the EAAT2 promoter, activates the NF-*κ*B signaling pathway, and elevates EAAT2 transcription [6, 40]. Our present results also showed that overexpression of Wnt1 increased the level of NF-*κ*B p-65 both in the cytoplasm and nucleus, indicative of the NF-*κ*B pathway activation. A series of studies have demonstrated that NF-*κ*B is involved in the protective effects of Wnt1. For example, Bournat et al. reported that Wnt1 promotes cell survival by activating NF-*κ*B in PC12 cells [41]; Shang and colleagues reported that microglial cell survival determined by Wnt1 during oxidative stress requires NF-*κ*B p65 [42]. The above results indicate that the crosstalk of the Wnt signal with the NF-*κ*B signal participates in the regulation of EAAT2 expression.

In conclusion, we found that 6-OHDA treatment decreased Wnt1 levels in U251 cells. Wnt1 promoted EAAT2 expression and mediated the cytoprotective effects of astrocytes on dopaminergic cells. Furthermore, NF-*κ*B might be involved in the regulation of EAAT2 by Wnt1.

## 5. Highlights

We conclude the following:

- 6-OHDA inhibits the expression of Wnt1, *β*-catenin, and EAAT2 in U251 cells
- Coculture with Wnt1-overexpressing U251 astrocytes attenuated the toxic effect of 6-OHDA on dopaminergic SH-SY5Y cells
- Wnt1 activated NF-*κ*B, promoted EAAT2 expression, and mediated the cytoprotective effects of astrocytes on dopaminergic cells

## Data Availability

The data used to support the findings of this study are included within the article.

## Abbreviations

6-OHDA: 6-Hydroxydopamine
DKK-1: Dickkopf-1
EAAT: Excitatory amino acid transporter
GAPDH: Glyceraldehyde-3-phosphate dehydrogenase
GLAST: Glutamate aspartate transporter
GLT-1: Glutamate transporter 1
GSK3*β*: Glycogen synthase kinase-3*β*
MPP+: 1-Methyl-4-phenylpyridinium ion
MPTP: 1-Methyl-4-phenyl-1,2,4,5-tetrahydropyridine
NF-*κ*B: Nuclear factor-*κ*B
PD: Parkinson's disease
SN: Substantia nigra.
